# Discovery of drugs to combat covid-19 inspired by traditional Chinese medicine

**DOI:** 10.1590/S0104-59702023000100010en

**Published:** 2023-04-03

**Authors:** Jianan Huang

**Affiliations:** i School of Social Sciences/Nanyang Technological University. Singapore. jhuang067@e.ntu.edu.sg

**Keywords:** coronavirus, pandemic, science, technology and society, ethnopharmacology, history of medicine, coronavirus, pandemia, ciencia, tecnología y sociedad, etnofarmacología, historia de la medicina

## Abstract

Contributions from traditional knowledge and history have proven useful in recent years to advance drug discovery. In response to the emergence of covid-19, scientists revisited traditional Chinese medicine. This source of inspiration for drugs to treat this new disease is described here at three different levels: traditional Chinese medicinal herbs, traditional Chinese medical formulas, and traditional Chinese medical texts. Drug discovery inspired by traditional Chinese medicine still faces serious resistance for various reasons, including its system of formulas and clinical trial design. A perspective that includes related issues would benefit the reasonable application of traditional knowledge in drug research and development.

## Resumen

Las contribuciones del conocimiento tradicional y la historia han demostrado ser útiles en los últimos años para avanzar en el descubrimiento de los medicamentos. En respuesta a la aparición de covid-19, los científicos revisaron la medicina tradicional china. Esta fuente de inspiración de los medicamentos para tratar esta nueva enfermedad se describe aquí en tres niveles diferentes: hierbas medicinales chinas tradicionales, fórmulas médicas chinas tradicionales y textos médicos chinos tradicionales. El descubrimiento de fármacos inspirado en la medicina tradicional china aún enfrenta serias resistencias por varias razones, incluido su sistema de fórmulas y el diseño de ensayos clínicos. Una perspectiva que incluya cuestiones conexas beneficiaría la aplicación razonable de los conocimientos tradicionales en la investigación y el desarrollo de fármacos.

## Traditional knowledge, contemporary medicine, and drug discovery

Historical records and lessons have made valuable contributions to advances in contemporary medicine (Kushner, 2008) as well as drug discovery (Holland, 1996). However, the most successful cases integrated ancient and modern medical knowledge that share the same knowledge system, which can be broadly described as evidence-based medicine. The benefits of traditional knowledge from systems that differ from contemporary medicine consequently remain controversial, especially in relatively technical fields such as drug discovery. According to Evans (2008), an invisible barrier exists between traditional knowledge and evidence-based medicine. This barrier may be even higher for traditional Chinese medicine (TCM), since this system is often considered experiential medicine (Zhan, 2014).

Because of the unique system that comprises biomedical research, clinical trials, and regulation, the process of drug discovery seems unfriendly to traditional medicine. The journal *Science* released three supplementary issues titled *The art and science of traditional medicine* during 2014-2015*,* with a special focus on traditional Chinese medicine. But even this level of recognition did not eliminate doubts among biological scientists: ultimately, controversies continue about the contemporary value of traditional medicine. Pharmacologists have similarly recognized very few cases of drug discovery from Ayurvedic medicine, despite significant investment by the Indian government in its Golden Triangle project to integrate bioscience, modern medicine, and traditional medicine (Patwardhan, Mashelkar, 2009). A similar phenomenon can also be seen with the Samoan traditional herbal medicine known as *matalafi* (Molimau-Samasoni et al., 2021).

## Experience and desire to revive the legacies of traditional Chinese medicine

Despite the above mentioned resistance, drug discoveries have repeatedly been inspired by Chinese medicine. In the 1940s, the Republic of China supported a national project related to the discovery of febrifugine (C16H19N3O3). The traditional herbal medicine *Dichroa febrifuga* (*changshan*) has been used in East Asia to treat malaria for nearly two thousand years. It was first recorded in the *Treatise on cold diseases and miscellaneous diseases* (*Shanghan zabing lun*) by Zhang Zhongjing (150/154-215/219). Inspired by ancient medical records, Chinese scientists discovered febrifugine from this plant (Jang et al., 1946), which may have been the first successful extension of a Chinese traditional drug into the western medical system (Lei, 1999).

The People’s Republic of China also launched a similar project in the 1970s, when a team led by Tu Youyou developed artemisinin (C15H22O5) from *Artemisia apiacea* (*qinghao*) (Tu et al., 1982). Tu drew her inspiration from a Taoist medical classic titled *Emergency preparedness prescriptions to keep at hand* (*Zhou hou bei ji fang*) written by Ge Hong (283-363) (Tu, 2011; Miller, Su, 2011). Tu won the Nobel prize in Physiology or Medicine in 2015, taking another step toward the dream to modernize Chinese medicine (Fu, 2017; Zhai, Wang, Li, 2016). Such endeavors continue in the new century; inspired by medical records written by Sun Simiao (581-682) and Li Shizhen (1518-1593), a team led by Zhu Chen applied arsenic trioxide (As_2_O_3_) to treat acute promyelocytic leukemia (Chen, Chen, 2017).

## Discovering drugs to treat covid-19 inspired by traditional Chinese medicine

The recent covid-19 pandemic drove scientists to revisit TCM; this system encourages them to seek inspiration above all from Chinese medicinal herbs. A networked pharmacological approach was developed to source Chinese medicinal herbs with potential to treat covid-19 and to investigate their underlying mechanisms (Pan et al., 2020). The initial results defined the top ten most widely used Chinese medicinal herbs, which include Glycyrrhizae Radix Et Rhizoma, Armeniacae Semen Amarum, Gypsum Fibrosum, Scutellariae Radix, Forsythiae Fructus, Poria, Ephedrae Herba, Citri Reticulatae Pericarpium, Pogostemonis Herba, and Lonicerae Japonicae Flos. This is a relatively established approach, with successful applications for many other diseases (Basu, Mallik, Mandal, 2017).

Further explorations have been inspired by the formulas in ancient Chinese medicine, rather than only herbal medicine and ethnopharmacology. The most successful cases are known as SYSF (*San yao san fang*, literally “three herbal formulas and three Chinese patent medicines”) drugs, which have been approved by the Chinese National Medical Products Administration (previously known as China FDA) between 2020 and 2021. A team from the Beijing University of Chinese Medicine also summarized potential anti-covid applications for the herbs and formulas recorded in the *Inner canon of the yellow emperor* (*Huang di nei jing*), a medical classic written in early China (Luo et al.*,* 2020). The extensive records of TCM formulas make it possible for contemporary researchers to pursue drug discovery based on traditional medicine rather than natural medicine.

**Figure 1:**
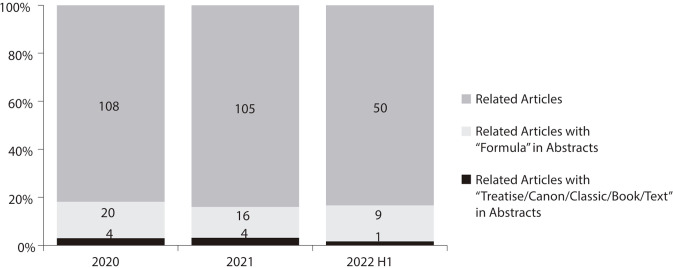
Three heterogeneous levels of covid-19 drug discovery inspired by TCM (Source: Web of Science; access on: 30 June 2022)

Other investigations have advanced in the discovery of drugs to combat covid-19 with inspiration from traditional Chinese medical texts and records, unlocking the potential of ancient medical treatises on TCM for contemporary medical therapies (Li, 2020; Wu, X. et al., 2021). Specifically with regard to covid-19, the *Treatise on cold diseases and miscellaneous diseases* and the *Treatise on differentiation and treatment of seasonal warm diseases* (*Wen bing tiao bian*) by Wu Jutong (1758-1836) have been highlighted (Wu, J. et al., 2021). While they essentially follow the methodology of modern biomedicine, they give more credit to ancient texts and records of Chinese medicine. Figure 1 depicts the results of a Web of Science search on covid-19 drug discovery inspired by TCM. The search formula used was: TI=(COVID OR coronavirus OR pandemic) AND TI=(“Chinese medicine” OR “Chinese herbal medicine” OR “TCM”) AND AB=(Unspecific/Formula/“Treatise OR Canon OR Classic OR Book OR Text”).

## Identification, modernization and resistance

Most drugs inspired by TCM to treat covid-19 are compound prescriptions. The best-known SYSF therapies usually contain over ten herbal medicines, and every herbal medicine contains two or more active ingredients. Notably, the mechanisms of action for these individual active ingredients generally have not been documented, even when these drugs are approved in specific countries such as China. Combination drugs or fixed-dose combinations can be utilized in contemporary biomedicine, but only when the mechanism of action for each individual drug in the combination therapy has been clearly explained. As a result, SYSF therapies have not been approved in health care systems dominated by modern biomedicine, such as those in the United States and Singapore.

Clinical trials present an additional challenge. At this time, most TCM drugs to treat covid-19 have not been subjected to serious, three-phase clinical trials. Some are “old drugs” with “new indications” for covid-19 that have earned the approval of China’s health authorities. This may be a common approach in TCM, but is not the best choice for achieving modernization and internationalization. Standardized drug discovery and development, in contrast, is generally a lengthy process. One example is YIV 906 (previously known as PHY-906), a four-herb Chinese medicine formula to treat liver cancer. Inspired by the *Treatise on cold diseases and miscellaneous diseases*, YIV 906 has been scrutinized for over ten years, from early research to Phase 2 clinical trials.

The unique knowledge system shaped by TCM has also encouraged resistance to big pharma in China and abroad, especially during the pandemic. The main drugs or candidates to treat covid-19 based on TCM were all developed by mid-sized pharmaceutical firms headquartered in mainland China, including Tianjin Chase Sun Pharmaceutical and Shijiazhuang Yiling Pharmaceutical. While multinational pharmaceutical corporations do have more advantages in terms of research and development, and capital availability, they tend not to have standalone sales teams for TCM and are not especially familiar with specialized regulatory policies. For this reason, big pharma did not play as large a role in China for treatment of covid-19 as it does for other infectious diseases.

Besides the resistance mentioned, newly emerging biological therapies and the biotechnology revolution also created challenges for the modernization of TCM. The success cases inspired by Chinese medicine mentioned above are all chemical therapies (febrifugine and artemisinin), which may face an increasing impact caused by the recent popularity of macro-molecular biological therapies. Against the backdrop of globalization and modernization, the future of alternative/complementary medicine remains uncertain.

## Final considerations

In comparison with the successful cases mentioned here, the drugs to treat covid-19 that were inspired by Chinese medicine still have a long road ahead. The models for drug discovery based on TCM (such as *changshan* and *qinghao*) are all single-molecule drugs with clear mechanisms of action. While contemporary biomedicine is in favor of combination drugs, the research process from single-molecule drugs to combination drugs represents a challenge. Unless the mechanisms of individual compounds can be explained in detail, direct applications of ancient compound prescriptions may not always be reasonable and acceptable by the public (Chen, Pei, Lu, 2013; Zhou, 2021).

**Figure 2:**
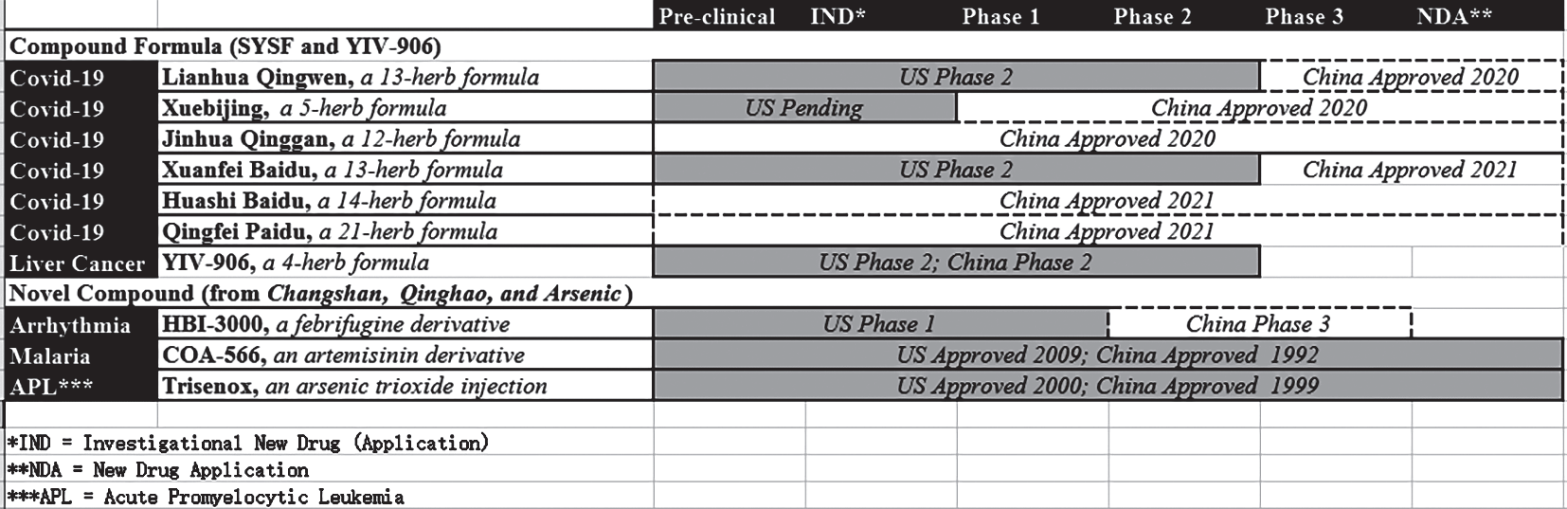
A landscape of drug pipelines inspired by traditional Chinese medicine

The century of efforts to discover drugs through Chinese medicine is reflected in China’s quest for a Nobel prize and the recent covid-19 crisis. Traditional medicine has not only transformed to fit into the western system, but also faced challenges and resistance. This brief sketch of recent developments in the applications of Chinese medicine for covid-19 drug discovery also reflects the long-term modernization of TCM resulting from successful research involving febrifugine (in the 1940s), artemisinin (in the 1970s), and many other molecules (Figure 2).
